# Evaluation of photoreceptor integrity in diabetic retinopathy using high-resolution optical coherence tomography

**DOI:** 10.1038/s41433-026-04353-z

**Published:** 2026-03-03

**Authors:** Kim Lien Huber, Dorota Himmel, Irene Steiner, Natasa Jeremic, Paul Widmann-Sedlnitzky, Heiko Stino, Laura Kunze, Gregor S. Reiter, Ursula Schmidt-Erfurth, Andreas Pollreisz

**Affiliations:** 1 Department of Ophthalmology, Medical University of Vienna, Vienna, Austria; 2 Center for Medical Data Science, Institute of Medical Statistics, Medical University of Vienna, Vienna, Austria

**Keywords:** Predictive markers, Retinal diseases

## Abstract

**Background:**

The relative ellipsoid zone reflectivity (rEZR) is a promising imaging biomarker for photoreceptor integrity, but its variation across diabetic retinopathy (DR) stages remains unclear. This study investigates rEZR changes across different DR severities using high-resolution optical coherence tomography (OCT) imaging.

**Methods:**

For this exploratory cross-sectional study, 64 eyes of 44 patients were included (17 eyes: no DR, 33 eyes: non-proliferative DR (NPDR), 14 eyes: proliferative DR (PDR)). Imaging was performed using high-resolution spectral-domain OCT (SPECTRALIS HighRes OCT, Heidelberg Engineering, axial resolution: 3 μm). rEZR (ratio between ellipsoid zone and external limiting membrane reflectivity) was measured at the foveola, within the Early Treatment Diabetic Retinopathy Study ETDRS grid (central 1 mm, inner ring, outer ring) and peripapillary. Linear mixed models assessed the association between rEZR, DR stage and visual acuity.

**Results:**

In the outer macular ring, mean rEZR was significantly lower in PDR compared to no DR (*p* = 0.005) and NPDR (*p* = 0.045). In the total area, mean rEZR was significantly lower in PDR compared to no DR (*p* = 0.016) and NPDR (*p* = 0.032). Peripapillary mean rEZR was significantly lower in NPDR compared to no DR (*p* = 0.013) and in PDR compared to no DR (*p* = 0.033). No significant group differences were observed in the foveola, central 1 mm or inner macular ring.

**Conclusion:**

Our data suggest that more advanced stages of DR are associated with a decrease in photoreceptor integrity, with statistically significant group differences predominantly observed in the outer macular regions and peripapillary.

## Introduction

The relative ellipsoid zone reflectivity (rEZR) has recently gained attention as a potential imaging marker for assessing photoreceptor integrity across different retinal diseases [1–5]. Early photoreceptor loss and outer retinal changes in hyperglycaemic mice have been described in histological ex vivo analyses, suggesting that photoreceptor degeneration may precede vascular abnormalities in the development of DR [6]. More recently, optical coherence tomography (OCT) studies in humans have confirmed that neurodegeneration is an early and potentially primary event in DR development [7, 8]. The integrity of the ellipsoid zone (EZ) is crucial for maintaining vision, and its disruption has been associated with visual impairment [9]. The first reflective band of the outer retina has been attributed to the external limiting membrane (ELM) representing the complex between Müller cells and photoreceptor neurons [10]. The second band has been assigned on spectral-domain and swept-source OCT images to the EZ of photoreceptors, which is densely packed with mitochondria [10, 11]. In this study, the use of SPECTRALIS High-Res OCT (Heidelberg Engineering GmbH, Heidelberg, Germany) with an axial resolution of less than 3 μm allows high-resolution analysis of retinal details. Studies have shown that diabetic patients without clinical signs of DR or mild non-proliferative DR present with reduced outer retinal reflectivity compared to healthy controls [12, 13]. This is the first study to investigate rEZR changes across different DR severities using high-resolution OCT imaging, offering insights into its potential as a quantitative OCT biomarker for disease staging in DR.

## Materials and methods

For this exploratory, cross-sectional study, patients with type 2 diabetes were recruited from the Department of Ophthalmology at the Medical University of Vienna. Routine clinical examination including best-correct visual acuity testing, slit-lamp biomicroscopy and medical history was taken from all included patients. After informed consent was given, patients were imaged with high-resolution spectral-domain (SD) OCT device (SPECTRALIS High-Res OCT, Heidelberg Engineering, Heidelberg, Germany). The device operates at a central wavelength of 850 nm and an axial resolution of 3 μm. OCT imaging protocol entailed two 30° × 5° horizontal and vertical scan patterns centred on the fovea with 48 sections. Automated real time was set to 16 frames. Furthermore, ultra-widefield colour fundus photography (CF) using either Zeiss Clarus 700 (Zeiss Meditec Inc., Dublin, USA) or Optos® California (Optos, Dunfermline, Scotland, UK) was taken from all included eyes. Retinal imaging was performed after pupil dilatation with 0.5% tropicamide eye drops.

Included were type 2 diabetic patients without clinical signs of DR, non-proliferative DR (NPDR) and proliferative DR (PDR). Exclusion criteria were refractive errors >6 dioptres of spherical equivalent, presence of DME (central subfield thickness over 250 μm), previous history of ocular inflammation or the presence of other retinal diseases. None of the patients had a known history of neurological disease. Furthermore, images with inadequate quality caused by motion artifacts, media opacities or with an image quality score (Q-score) under 25 were excluded from further analysis. The Q-score is integrated into the device to assess signal to noise ratio. The study was conducted following the endorsement of the ethics committee (number 1548/2021) of the Medical University of Vienna in alignment with the Declaration of Helsinki. The sample size consists of all eligible patients during the recruiting period, no sample size calculation was performed due to the exploratory nature of this analysis.

### Image analysis

DR severity level was assessed by two expert graders (HS, LK) on colour fundus photography based on the “Diabetic Retinopathy Disease Severity Scale” (DRSS) and proliferative DR was confirmed by fluorescein angiography (Optos® California, Dunfermline, Scotland, UK). In case of discrepancies between graders, the final DR stage was determined by a senior grader and retinal specialist (AP). OCT scans were exported from the device as TIFF images to Fiji [14] and graders were blinded for DR stage for further analysis. Regions of interest (ROIs) were placed on each scan by one grader and then reviewed and confirmed by a second grader. Once the locations were agreed upon, reflectivity values of EZ and ELM were analysed using “plot profile” function in Fiji. Three consecutive fovea-centred horizontal and vertical OCT B-scans per eye were evaluated. Measurements were taken from the foveola and ROIs were set at nasal (n), temporal (t), superior (s) and inferior (i) positions, each at 500 μm, 1000 μm and 2000 μm eccentricity from the foveola. Additionally, in horizontal B-Scans, measurements were taken at the peripapillary region with a distance of 3000μm from the foveola. The measurements in the subareas were combined for further analysis to represent the standard ETDRS grid: central 1 mm disc (mean of n_500μm, t_500μm, s_500μm, i_500μm), inner ring (mean of n_1000μm, t_1000μm, s_1000μm, i_1000μm) and outer ring (mean of n_2000μm, t_2000μm, s_2000μm, i_2000μm). Analogously, subareas were combined for analysis of quadrants (e.g. nasal quadrant: mean of n_500µm, n_1000µm, n_2000µm). The ROI was set at 15 pixel width and 40 pixel height. In case of vessel shadows or disruption of retinal layers due to pathologic signs of DR, ROI was shifted by 100 μm to the nearest area with continuous retinal layers. If no continuous retinal layers were available within 100 μm eccentricity, the affected retinal zone was excluded from further analysis to ensure comparable locations across eyes.

**Fig. 1:**
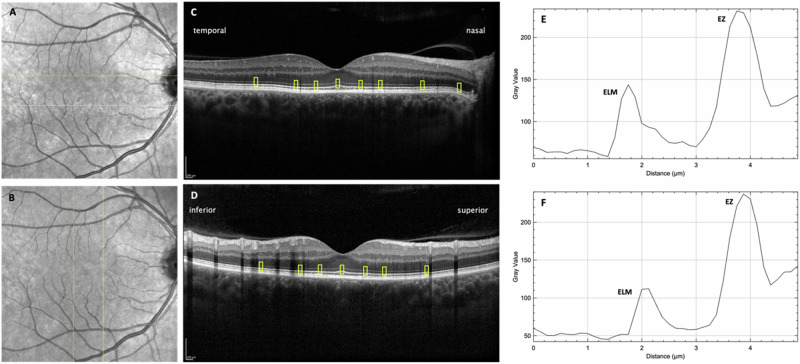
Example of a 61-year-old male patient with mild diabetic retinopathy case. **A**, **B** Infrared fundus images with yellow lines indicating the scanned areas. **C** Horizontal and **D** vertical OCT B-scans with yellow boxes marking the regions used for retinal reflectivity measurements at various distances from the foveal centre (foveola, 500 µm, 1000 µm, and 2000 µm) and at the peripapillary region in the horizontal scan. Representative reflectivity profiles of the horizontal (**E**) and vertical scans (**F**) are shown. The first peak corresponds to the external limiting membrane (ELM), and the second peak to the ellipsoid zone (EZ). Pixel intensity values range from 0 (black) to 255 (white) on a grayscale.

It was assumed that rEZR values of the subareas are similar within rings, but different within quadrants. Hence, for the combination of subareas within rings, the mean over all valid observations was taken, while for the combination of subareas within quadrants, the mean was only calculated if the rEZR value was available in all subareas, otherwise set to missing. rEZR in the total area corresponds to the mean value over rEZR in the central 1 mm disc, inner ring and outer ring. The peak intensity of the first and second reflective band was documented, corresponding to the EZ and ELM. The reflectivity was recorded from 0 to 255 in grey value. Figure 1 shows OCT B-Scans with overlaid ROIs and example reflectivity profiles of the ELM and EZ. As previously described in the literature, the rEZR is calculated as the ratio of EZ to ELM to account for variations in OCT brightness [1, 2, 5]. The ELM was chosen for reference because it is a nonneural layer with a relative constant signal intensity across the eccentricity [10, 15, 16].

### Statistical analysis

Metric variables were summarised using the mean and standard deviation (SD) if approximately normally distributed or median and interquartile range (IQR) otherwise. Absolute frequencies were reported for qualitative variables. The endpoint was rEZR in different locations. For each location, a linear mixed-effects model was fitted to assess the impact of the independent variable DR severity (categorical, 3 groups) on the endpoint ellipsoid zone reflectivity, with disease duration (years), age (years), gender (reference = male) and eye side (reference = right eye) included as confounder variables in the model. Patient-ID was taken as random factor to account for intra-subject correlation due to measurements on both eyes. In sum, 10 mixed models were calculated. For DR severity, an overall test was performed (H0: equal means in all groups) using an F-test (type III). In case of a significant result, pairwise comparisons were conducted (R-package emmeans_1.10.4, R-function emmeans, adjust = “none”) without adjustment for multiple testing due to the exploratory character of the study and the limited number of groups.

To compare rEZR between the rings, a mixed model was calculated with rEZR as dependent variable. Independent variables were ring (3 rings), DR severity (categorical, 3 groups), age (years), disease duration (years), gender (reference = male), eye side (reference = right eye) and the interaction between DR severity and ring. Patient-ID was included as random effect to account for within-subject correlation, and additionally, the nested factor “eye within patient” was included to consider that three measurements per eye were analysed. If the interaction term revealed a *p*-value < 0.05, pairwise comparisons of rings were conducted based on the mixed model using Tukey adjustment within each DR stage (R-function emmeans, adjust = “tukey”). To analyse the association between visual acuity (logMAR) and rEZR, a mixed model was calculated. Visual acuity was the dependent variable, rEZR in the total area (model 1) or the fovea (model 2) was the independent variable. Patient-ID was included as random factor. In a further step, we included age (years), disease duration (years) and DR stage (categorical, 3 groups) as additional independent variables.

Mixed models were calculated using the R-function lmer (R-packages lme4_1.1-35.3 and lmerTest_3.1-3). Statistical analyses were carried out with R 4.4.0 [17]. The significance level has been set to 0.05. Note that the analyses are exploratory.

## Results

84 eyes of 55 patients were recruited for this study. Four eyes were excluded due to motion artifacts or an OCT quality-score under 25, 5 eyes due to diabetic macular oedema and 11 eyes were excluded due to quiescent proliferative DR. Included were 64 eyes (33 right eyes) of 44 patients (12 female). Seventeen eyes without clinically visible DR, 8 eyes with mild, 9 eyes with moderate and 16 eyes with severe DR were included in the NPDR group and 14 eyes in the PDR group. Because of the limited number of eyes with mild, moderate and severe NPDR, these were pooled into a single NPDR category for statistical analyses. Mean image quality score was 33 ± 4. Patient demographics are summarised in Table 1.

**Table 1:** Patient characteristics per diabetic retinopathy group: without clinical signs of diabetic retinopathy (no DR), non-proliferative diabetic retinopathy (NPDR) and proliferative diabetic retinopathy (PDR).

	No DR *n* = 17	NPDR *n* = 33	PDR *n* = 14
Age, years	60.3 ± 9.1	65.1 ± 10.2	59.4 ± 6.9
DM duration, years	9.9 ± 5.9 missing = 4	17.3 ± 7.5 missing = 3	19.6 ± 10.7 missing = 2
HbA1c, %	7.3 ± 1.0 missing = 4	7.1 ± 1.3 missing = 11	7.1 ± 1.7 missing = 6
BCVA, logMAR	−0.01 (−0.1; 0)	0 (–0.1; 0.1)	0.05 (0; 0.18)
Eye side: right eyes	9 (53%)	17 (52%)	7 (50%)
Treatment-naive eyes	17 (100%)	22 (67%)	5 (36%)
Prior laser photocoagulation	0	2 (27%)	8 (57%)
Prior anti-VEGF injections	0	9 (27%)	9 (64%)

*BCVA* best corrected visual acuity, *n* number of eyes, *DM* diabetes mellitus.

Metric variables are presented as mean ± standard deviation if approximately normally distributed and as median (first quartile; third quartile) otherwise. Categorical data are summarised as counts and absolute frequency. Note that PDR patients might have received >1 treatment option per eye.

### Comparison of rEZR between DR stages

**Fig. 2:**
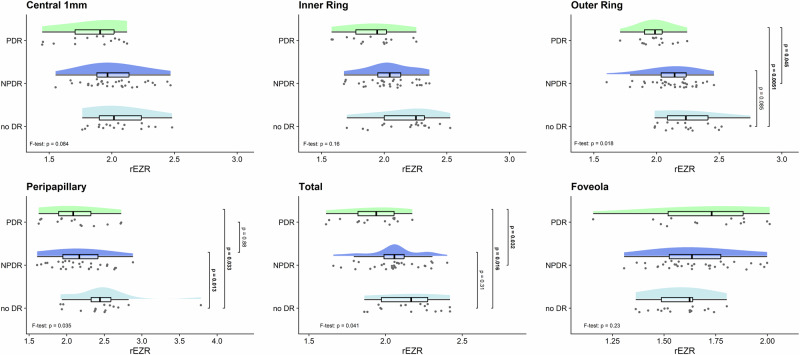
Raincloud plots show relative ellipsoid zone reflectivity (rEZR) values for different retinal locations (as indicated in the titles) across eyes with no diabetic retinopathy (No DR), non-proliferative DR (NPDR), and proliferative DR (PDR). The figure combines a density plot (“cloud”) showing the smoothed distribution of the data, a boxplot and the raw data (“rain”) jittered below the density to display individual observations. The *F*-test revealed a *p*-value < 0.05 in the outer ring, peripapillary and total area. The *p*-values of the pairwise comparisons are shown next to the raincloud plot. *P*-values < 0.05 are displayed in bold.

Statistically significant group differences were observed in the outer ring, the peripapillary region as well as in the total area (*F*-tests: *p* = 0.018, *p* = 0.035 and *p* = 0.041, respectively). In the outer macular ring, mean rEZR was significantly lower in PDR compared to no DR (*p* = 0.005), and also lower in PDR compared to NPDR (*p* = 0.045). In the total area, rEZR was on average significantly lower in PDR compared to no DR (*p* = 0.016), and in PDR compared to NPDR (*p* = 0.032). In the peripapillary region, mean rEZR was significantly lower in NPDR compared to no DR (*p* = 0.013), as well as in PDR compared to no DR (*p* = 0.033). No significant differences between DR stages were observed in the foveolar (*F*-test: *p* = 0.23), central 1 mm (*F*-test: *p* = 0.084), or inner macular ring (*F*-test: *p* = 0.16). Group comparisons are adjusted for age, gender, eye (right/left) and disease duration. Table 2 shows the results of the linear mixed models for each location. Figure 2. shows raincloud plots illustrating the differences in rEZR between the DR stage groups and retinal location.

**Table 2:** The table summarises the results of linear mixed model analyses assessing the association between the relative ellipsoid zone reflectivity and diabetic retinopathy stage across different retinal locations.

Retinal localisation	DR stage	Estimate [95% CI]	*p*-value	*F*-test	*n*
Foveola	DR groups			0.23	55 (38)
Central 1 mm	DR groups			0.084	55 (38)
Inner ring	DR groups			0.16	55 (38)
Outer ring	NPDR vs no DR	−0.128 [−0.275; 0.018]	0.085	**0.018**	55 (38)
	PDR vs no DR	−0.283 [−0.476; −0.091]	**0.005**		
	PDR vs NPDR	−0.155 [−0.306; −0.004]	**0.045**		
Total	NPDR vs no DR	−0.074 [−0.219; 0.072]	0.31	**0.041**	55 (38)
	PDR vs no DR	−0.246 [−0.442; −0.049]	**0.016**		
	PDR vs NPDR	−0.172 [−0.328; −0.015]	**0.032**		
Peripapillary	NPDR vs no DR	−0.363 [−0.643; −0.082]	**0.013**	**0.035**	50 (34)
	PDR vs no DR	−0.384 [−0.733; −0.034]	**0.033**		
	PDR vs NPDR	−0.021 [−0.301; 0.259]	0.88		

*DR* diabetic retinopathy, *CI* confidence interval, *NPDR* non-proliferative DR, *PDR* proliferative DR.

For each location, the *p*-value of the *F*-tests are shown. If the *F*-test was statistically significant, additionally, the results of pairwise comparisons between disease stages (no DR, NPDR, PDR) are shown, including the estimated effect sizes with 95% confidence intervals, *p*-values (not adjusted for multiplicity), and sample size (*n*) Associations are adjusted for age, gender, eye side and disease duration. Significant overall group effects were observed in the outer macular ring (*F*-test: *p* = 0.018), total area (*p* = 0.041), and peripapillary region (*p* = 0.035). Numbers in bold indicate statistically significant *p*-values (*p* < 0.05).

In the supplementary material (Supplementary Table S1), the results of the mixed model analyses are presented, including the independent variables age, disease duration, gender and eye (right/left), as well as the analysis of rEZR across the nasal, temporal, superior and inferior quadrants. No significant differences in rEZR were found between the quadrants across DR stage. In the Supplementary Table S2 descriptive statistics of rEZR in the different regions can be found.

### Comparison of rEZR between retinal locations

Mixed model analysis revealed a significant interaction between DR group and ring (*p* = 0.043). Hence, Tukey tests were calculated for comparison of rings within each DR stage. In eyes with no DR, mean rEZR significantly differed between all rings: central vs. inner ring (estimate [Tukey adjusted 95% CI]: −0.148 [−0.26; −0.036], *p* = 0.0062), central vs. outer ring (−0.278 [−0.390; −0.166], *p* < 0.0001) and inner vs. outer ring (−0.130 [−0.242; −0.017], *p* = 0.019).

In NPDR eyes, mean rEZR was significantly lower in the central 1 mm ring compared to the outer ring (−0.110 [−0.184; −0.037), *p* = 0.002) and the inner ring compared to the outer ring (−0.094 [−0.168; −0.020], *p* = 0.009).

In PDR eyes, only the comparison between central 1 mm versus the outer ring was significant (−0.173 [−0.289; −0.056], *p* = 0.002). These differences are illustrated graphically in Fig. 2. Details of the mixed models are provided in the Supplementary Tables S3, S4.

### Association between visual acuity and rEZR

In the univariable analysis, the association between visual acuity and rEZR in the total area was significantly negative (*p* = 0.035). When adjusting for age, disease duration and DR stage, the association with rEZR in the total area was not statistically significant (*p* = 0.31). The association of visual acuity and rEZR in the fovea was not statistically significant (*p* = 0.23 and *p* = 0.54). Results of the mixed models are shown in Table 3.

**Table 3:** Results of the mixed models with visual acuity (logMAR) as dependent variable and relative ellipsoid zone reflectivity (rEZR) in the total area (Model 1) and in the fovea (Model 2).

	Variable	Univariable model	*p*-value	*n*	Multivariable model	*p*-value	*n*
		estimate [95% CI]			estimate [95% CI]		
Model 1	rEZR Total	−0.171 [−0.326; −0.015]	0.035	64 (45)	−0.095 [−0.275; 0.085]	0.31	55 (38)
	Age (years)				0.008 [0.003; 0.013]	0.007	
	Disease duration (years)				−0.006 [−0.012; 0.0002]	0.07	
	DR stage					0.07	
Model 2	rEZR Fovea	0.068 [−0.041; 0.176]	0.23	64 (45)	0.04 [−0.085; 0.164]	0.54	55 (38)
	Age (years)				0.008 [0.003; 0.014]	0.007	
	Disease duration (years)				−0.005 [−0.012; 0.001]	0.12	
	DR stage					0.044	

On the left side, the results of the univariable models are shown, on the right side the results of the multivariable models. The *p*-values of DR-stage were derived from *F*-tests.

*n* number of eyes (number of patients), *CI* confidence interval.

## Discussion

In this study, we quantitatively assessed photoreceptor integrity across different stages of DR by measuring the rEZR. Our findings demonstrate a progressive reduction of rEZR with increasing DR severity, particularly in the outer macular ring and peripapillary region.

The use of high-resolution OCT imaging in this study provides advantages over standard OCT imaging modalities, as prior studies demonstrated. Frank et al. showed an improved delineation of outer retinal layers in eyes with age-related macular degeneration images with the Spectralis High-Res OCT (axial resolution: 3 μm, lateral resolution: 14 μm, central wavelength: 853 nm) compared to Spectralis SD-OCT (axial resolution: 7 μm, lateral resolution: 14 μm, central wavelength: 880 nm) [18]. Another comparative study between the two devices demonstrated that the high-resolution OCT enhanced both inter-reader and intra-reader annotation results in retinal layer segmentation [19].

The rEZR (ratio of EZ/ELM reflectivity) enables quantitative assessment of photoreceptor integrity. This emerging OCT-based approach has been established in recent studies of age-related macular degeneration and macular telangiectasia, where it has proven to be a reliable imaging biomarker for detecting photoreceptor damage, correlating with both disease severity and functional decline [1, 2, 5, 13, 20]. However, methods for analysing ellipsoid zone reflectivity in diabetic patients vary in previous studies and are limited to no DR and NPDR cases. Toprak et al. were the first to analyse retinal layer OCT reflectivity in mild NPDR and found reduced photoreceptor inner segment ellipsoid layer reflectivity compared to healthy controls. Notably, reflectivity measurements were restricted to the fovea, using the RPE as the reference layer [13]. Zhang et al. reported early EZ reflectivity loss in diabetic eyes without DR compared to healthy controls, correlating with reduced vascular density and suggesting its potential as a marker of early photoreceptor degeneration. They assessed relative EZ reflectivity using the RPE as the reference layer with measurement locations comparable to the regions analysed in our study [12]. Consistent with our findings, no significant changes were observed at the foveola. However, in contrast to our results, they reported significant reflectivity reductions beyond 500 μm eccentricity from the foveal centre, whereas in our study, changes reached statistical significance only at 2000 μm foveal eccentricity and 3000 μm (peripapillary) [12]. Borrelli et al. found that NPDR eyes had a lower EZ reflectivity than healthy controls in the 3 × 3 mm macular EZ slab [21]. Extending these findings across all DR stages, we demonstrate a progressive decline in rEZR with increasing disease severity, most pronounced in the outer macular and peripapillary regions. In contrast to Parravano et al., who observed no difference between no DR and NPDR in type 1 diabetes [22], we detected a significant peripapillary rEZR reduction. This discrepancy may be attributed to differences in disease type (type 2 vs type 1 diabetes), reflectivity quantification methods or the sample size of the investigations. While Parravano et al. and Borelli et al. quantified brightness on en face EZ slabs from OCT images, we applied a B-scan-based rEZR approach, which has been validated in prior studies [1, 2, 5]. To account for variations in OCT signal intensity, EZ reflectivity is normalised to the ELM, a nonneural layer with a relative constant signal intensity across the eccentricity [15, 16]. These differences in EZ quantification highlight the need for standardised metrics in OCT-based imaging biomarkers and may explain variation across studies.

In the univariable model, we observed a negative correlation between VA and rEZR in the total area, however this association lost statistical significance when adjusting for age, DR stage and disease duration. Zhang et al. similarly described negative correlations between VA and EZ reflectivity at multiple parafoveal locations, but they did not account for confounding variables [12]. Visual acuity testing by autorefraction or ETDRS charts primarily reflects central, high-contrast cone function and may not capture the localised photoreceptor changes that rEZR detects. Future studies should therefore incorporate more sensitive functional tests such as low luminance VA, flicker electroretinography, contrast sensitivity or microperimetry to better delineate structure-function relationships and assess whether rEZR can serve as a biomarker for visual dysfunction in DR [23, 24].

The reflectivity signal of the EZ when imaged with SD-OCT may originate from the ellipsoid components of photoreceptor inner segments, which are densely packed with mitochondria, making reduced EZ reflectivity a potential indicator for photoreceptor disorganisation or damage [10, 16]. In DR these changes may stem from hyperglycaemia-induced mitochondrial dysfunction and oxidative stress, both central drivers of outer retinal degeneration [25]. Retinal neurodegenerative changes, such as photoreceptor apoptosis and glial activation, occur early in DR, often before microvascular abnormalities are visible [7, 22, 26]. Müller glial dysfunction, impaired neurovascular coupling, inflammation and pericyte loss further compromise photoreceptor health. Photoreceptor injury may then worsen vascular damage by releasing reactive oxygen species and proinflammatory cytokines [8, 27, 28].

Our analysis showed that rEZR significantly varies across different foveal eccentricities with the highest reflectivity observed in the perifoveal region with a decline towards the fovea. This pattern may reflect regional differences in mitochondrial distribution within the EZ, with increasing ellipsoid volume at greater eccentricities from the foveal centre [4, 29].

Furthermore, rod density in the perifoveal region is substantially higher than cone density, and Le et al. have shown that early DR is characterised by predominant rod photoreceptor damage [30].

The fovea may benefit from more robust metabolic support due to its dual vascular supply from the surrounding retinal circulation and the choriocapillaris [21, 31]. Vascular factors may therefore contribute to regional differences in EZ reflectivity. Borrelli et al. demonstrated that reduced EZ reflectivity in NPDR was associated with decreased choriocapillaris perfusion, suggesting that impaired choriocapillaris flow affects photoreceptor integrity and contributes to EZ reflectivity loss [21].

Limitations of this study include its cross-sectional design and the limited sample size, which preclude longitudinal follow-up and the assessment of causal effects over time. While we adjusted for key covariates (age, gender, disease duration, eye side), residual confounders may remain. Lastly, some eyes were previously treated with laser photocoagulation and intravitreal anti-VEGF injections, which could affect the observed rEZR signal intensity. Because of the small sample size and heterogenous treatment histories, we did not adjust for these effects and our findings should therefore be interpreted with caution.

## Conclusion

Standardised rEZR measurements on OCT scans across different macular eccentricities and in the peripapillary region may serve as potential biomarkers of photoreceptor damage in DR. Our data suggest that advanced stages of DR are associated with a more pronounced reduction of the rEZR, particularly in the outer macular and peripapillary regions.

## Summary

### What was known before

- The relative ellipsoid zone reflectivity (rEZR) is an emerging OCT-based biomarker for photoreceptor integrity.
- Reduced outer retinal reflectivity has been described in diabetic patients without clinical signs of diabetic retinopathy (DR) compared to healthy controls.

### What this study adds

- This is the first study to evaluate rEZR across different DR severities.
- rEZR reduction was most pronounced in the outer macular and peripapillary regions, particularly in proliferative DR.
- These findings support rEZR as a potential quantitative biomarker for photoreceptor integrity and disease staging in DR.

## Supplementary information

Supplemental Material

## Data Availability

The dataset generated and analysed during the current study is available from the corresponding author on reasonable request.
