# Supplementary material for: Evaluation of photoreceptor integrity in diabetic retinopathy using high-resolution optical coherence tomography
Source: Eye (Lond). 2026 Mar 3;40(8):1121–7. doi: 10.1038/s41433-026-04353-z (PMC13194956; doi:10.1038/s41433-026-04353-z)
Supplement: Supplementary file 1 — Supplemental Material [file 41433_2026_4353_MOESM1_ESM.docx]

**Evaluation of photoreceptor integrity in diabetic retinopathy using high-resolution optical coherence tomography**

**SUPPLEMENTAL MATERIAL**

| **Location** | **Variable** | **Estimate [95% CI]** | **p-value** | **F-test** | **n** |
| --- | --- | --- | --- | --- | --- |
| Central 1 mm | DR stage |  |  | 0.084 | 55 (38) |
|  | Gender | -0.023 [-0.202; 0.157] | 0.81 |  |  |
|  | Age | -0.002 [-0.012; 0.008] | 0.72 |  |  |
|  | Disease duration | -0.001 [-0.012; 0.01] | 0.85 |  |  |
|  | Eye | -0.011 [-0.092; 0.07] | 0.79 |  |  |
| Inner ring | DR stage |  |  | 0.16 | 55 (38) |
|  | Gender | 0.026 [-0.133; 0.185] | 0.75 |  |  |
|  | Age | -0.003 [-0.012; 0.006] | 0.49 |  |  |
|  | Disease duration | -0.005 [-0.015; 0.005] | 0.31 |  |  |
|  | Eye | -0.04 [-0.111; 0.03] | 0.28 |  |  |
| Outer ring | NPDR vs. No DR | -0.128 [-0.275; 0.018] | 0.085 | 0.018* | 55 (38) |
|  | PDR vs. No DR | -0.283 [-0.476; -0.091] | 0.005* |  |  |
|  | PDR vs. NPDR | -0.155 [-0.306; -0.004] | 0.045* |  |  |
|  | Gender | 0.151 [0.015; 0.287] | 0.038 |  |  |
|  | Age | -0.008 [-0.016; -0.001] | 0.039 |  |  |
|  | Disease duration | -0.001 [-0.01; 0.007] | 0.79 |  |  |
|  | Eye | -0.054 [-0.123; 0.016] | 0.15 |  |  |
| Superior | DR stage |  |  | 0.08 | 53 (38) |
|  | Gender | 0.023 [-0.164; 0.211] | 0.81 |  |  |
|  | Age | -0.002 [-0.012; 0.008] | 0.7 |  |  |
|  | Disease duration | 0 [-0.012; 0.011] | 0.96 |  |  |
|  | Eye | -0.014 [-0.11; 0.082] | 0.78 |  |  |
| Temporal | DR stage |  |  | 0.13 | 54 (37) |
|  | Gender | 0.04 [-0.114; 0.194] | 0.62 |  |  |
|  | Age | -0.004 [-0.012; 0.005] | 0.38 |  |  |
|  | Disease duration | -0.01 [-0.02; 0] | 0.052 |  |  |
|  | Eye | -0.037 [-0.095; 0.022] | 0.23 |  |  |
| Nasal | DR stage |  |  | 0.2 | 52 (36) |
|  | Gender | 0.144 [-0.041; 0.33] | 0.14 |  |  |
|  | Age | -0.005 [-0.015; 0.006] | 0.37 |  |  |
|  | Disease duration | -0.005 [-0.016; 0.007] | 0.44 |  |  |
|  | Eye | -0.058 [-0.173; 0.056] | 0.33 |  |  |
| Inferior | DR stage |  |  | 0.24 | 51 (34) |
|  | Gender | 0.016 [-0.143; 0.176] | 0.84 |  |  |
|  | Age | -0.004 [-0.013; 0.005] | 0.38 |  |  |
|  | Disease duration | 0.002 [-0.007; 0.011] | 0.65 |  |  |
|  | Eye | -0.061 [-0.174; 0.052] | 0.3 |  |  |
| Total | NPDR vs. No DR | -0.074 [-0.219; 0.072] | 0.31 | 0.041* | 55 (38) |
|  | PDR vs. No DR | -0.246 [-0.442; -0.049] | 0.016* |  |  |
|  | PDR vs. NPDR | -0.172 [-0.328; -0.015] | 0.032* |  |  |
|  | Gender | 0.049 [-0.093; 0.192] | 0.5 |  |  |
|  | Age | -0.004 [-0.012; 0.003] | 0.27 |  |  |
|  | Disease duration | -0.002 [-0.011; 0.006] | 0.58 |  |  |
|  | Eye | -0.034 [-0.093; 0.026] | 0.28 |  |  |
| Foveola | DR stage |  |  | 0.23 | 55 (38) |
|  | Gender | 0.067 [-0.072; 0.207] | 0.35 |  |  |
|  | Age | -0.002 [-0.01; 0.005] | 0.53 |  |  |
|  | Disease duration | -0.001 [-0.01; 0.007] | 0.74 |  |  |
|  | Eye | -0.066 [-0.153; 0.022] | 0.15 |  |  |
| Peripapillary | NPDR vs. No DR | -0.363 [-0.643; -0.082] | 0.013* | 0.035* | 50 (34) |
|  | PDR vs. No DR | -0.384 [-0.733; -0.034] | 0.033* |  |  |
|  | PDR vs. NPDR | -0.021 [-0.301; 0.259] | 0.88 |  |  |
|  | Gender | 0.316 [0.067; 0.566] | 0.02 |  |  |
|  | Age | -0.009 [-0.025; 0.006] | 0.25 |  |  |
|  | Disease duration | -0.001 [-0.015; 0.014] | 0.93 |  |  |
|  | Eye | -0.177 [-0.337; -0.017] | 0.039 |  |  |

**Table S1**: Results of mixed models analysing the association between rEZR and diabetic retinopathy stage across different retinal locations, adjusted for gender (reference = male), age, disease duration and eye side (reference = right eye). Variables superior, inferior, temporal and nasal area corresponds to the mean of the three rings (e.g. superior corresponds to the mean of superior 500µm, superior 1000µm and superior 2000µm). Shown are models estimated with 95% confidence interval and p-valued for fixed effects. For disease group, the F-test p-values are reported. Additionally, pairwise comparisons between groups (not adjusted for multiplicity) are reported if the F-test was statistically significant.

| **Variable** | **no DR (17 eyes)** | **NPDR (33 eyes)** | **PDR (14 eyes)** |
| --- | --- | --- | --- |
| central 1mm | 2.05 ± 0.21 | 2.01 ± 0.22 | 1.84 ± 0.23 |
| Inner Ring | 2.17 ± 0.23 | 2.04 ± 0.19 | 1.91 ± 0.21 |
| Outer Ring | 2.26 ± 0.21 | 2.12 ± 0.19 | 1.99 ± 0.14 |
| Total | 2.16 ± 0.18 | 2.05 ± 0.17 | 1.91 ± 0.18 |
| Foveola | 1.58 ± 0.13 | 1.65 ± 0.18 | 1.69 ± 0.24 |
| Peripapillary | 2.47 ± 0.44 (1 missing) | 2.17 ± 0.33 (4 missing) | 2.11 ± 0.37 |

**Table S2**: Descriptive statistics of rEZR in different areas. The table shows mean ± SD.

| Variable | p-value |
| --- | --- |
| DR group | 0.041 |
| Ring | < 0.001 |
| Gender | 0.5 |
| Age | 0.27 |
| Disease duration | 0.58 |
| Eye | 0.28 |
| DR_groups:Ring | 0.043 |

**Table S3:** Results of the F-tests, mixed model with rEZR as dependent variable, comparison of rings (Tukey tests).

| **DR stage** |  | **estimate** | **adjusted Lower-CL** | **adjusted Upper-CL** | **adjusted p-value** |
| --- | --- | --- | --- | --- | --- |
| No DR | central 1 mm vs. inner ring | -0.148 | -0.260 | -0.036 | 0.006 |
|  | central 1 mm vs. outer ring | -0.278 | -0.390 | -0.166 | < 0.001 |
|  | inner ring vs. outer ring | -0.130 | -0.242 | -0.017 | 0.019 |
| NPDR | central 1 mm vs. inner ring | -0.016 | -0.090 | 0.058 | 0.86 |
|  | central 1 mm vs. outer ring | -0.110 | -0.184 | -0.037 | 0.002 |
|  | inner ring vs. outer ring | -0.094 | -0.168 | -0.020 | 0.009 |
| PDR | central 1 mm vs. inner ring | -0.094 | -0.210 | 0.023 | 0.14 |
|  | central 1 mm vs. outer ring | -0.173 | -0.289 | -0.056 | 0.002 |
|  | inner ring vs. outer ring | -0.079 | -0.196 | 0.038 | 0.25 |

**Table S4:** Pairwise comparison of rings for each disease group separately.
